# A remaining useful life prediction method based on PSR-former

**DOI:** 10.1038/s41598-022-22941-3

**Published:** 2022-10-25

**Authors:** Huang Zhang, Shuyou Zhang, Lemiao Qiu, Yiming Zhang, Yang Wang, Zili Wang, Gaopeng Yang

**Affiliations:** 1 The State Key Laboratory of Fluid Power and Mechatronic System, Zhejiang University, Hangzhou, 310027 China; 2 Engineering Research Center for Design Engineering and Digital Twin of Zhejiang Province, Hangzhou, 310027 China

**Keywords:** Mechanical engineering, Computational science

## Abstract

The non-linear and non-stationary vibration data generated by rotating machines can be used to analyze various fault conditions for predicting the remaining useful life(RUL). It offers great help to make prognostic and health management(PHM) develop. However, the complexity of the mechanical working environment makes the vibration data collected easily affected, so it is hard to form an appropriate health index(HI) to predict the RUL. In this paper, a PSR-former model is proposed including a Phase space reconstruction(PSR) layer and a Transformer layer. The PSR layer is utilized as an embedding to deepen the understanding of vibration data after feature fusion. In the Transformer layer, an attention mechanism is adopted to give different assignments, and a layer-hopping connection is used to accelerate the convergence and make the structure more stable. The effectiveness of the proposed method is validated through the Intelligent Maintenance Systems (IMS) bearing dataset. Through analysis, the prediction accuracy is judged by the parameter RMSE which is 1.0311. Some state-of-art methods such as LSTM, GRU, and CNN were also analyzed on the same dataset to compare. The result indicates that the proposed method can effectively establish a precise model for RUL predictions.

## Introduction

Mechanical vibration is a common phenomenon in the operation of industrial equipment. With the increase of equipment service time, various wear and defects will gradually form on internal parts, resulting in equipment performance degradation, thus affecting the service life of the equipment. Bearing, as the core component of most rotating machines, once fails, the equipment will be affected or even collapsed, making it difficult to maintain the prognostic and health management (PHM)^1^ of the equipment which is a task for real-time equipment operation monitoring. This information including the equipment operation status will be reflected in the vibration data through abnormal fluctuations. Therefore, PHM needs to analyze the bearing vibration data and predict the remaining useful life(RUL) that enables the service time of the equipment to be described quantitatively^2^ which is conducive to reducing costs and improving production efficiency.

PHM driven by data to predict RUL is inseparable from time analysis methods^3^. The numerical calculation method^4^, data-driven method^5,6^, and data-model combination method^7,8^ are the common methods for RUL prediction in time analysis. Among the methods using the modeling approach for prediction, Gabelli et al.^9^ predicted the RUL based on the rolling contact fatigue theory study by expressing the survival probability of the raceway surface as the basic life equation. In data-model combination, Qin et al.^7^ estimated the size of the defects by measuring the signals to reveal the evolution law of the defects in time and formed the bearing dynamics model. Since the model-based and data-model-based approaches require an explicit and comprehensive analytical model to reflect the bearing degradation process, it is difficult to achieve in practical production, making the physical model hard to describe complex systems. However, the data-driven approach allows the model to learn degradation patterns directly from the data collected by sensors to reduce the dependence on the physical knowledge background. Meanwhile, as the era of big data has led to the development of data-driven approaches ^10^, a large amount of industrial data can be collected, so data-driven approaches are gradually becoming dominant^11^.

The Recurrent Neural Network (RNN) has a unique recursive structure, which is conducive to extracting information from time series, so it is gradually applied to RUL prediction^12^. However, with long-term iteration, the information will be blurred gradually, and the gradient will disappear or explode. To solve the problem, Long short-term memory (LSTM) was proposed. LSTM ^13^ adds the "gate" structure to enhance the ability of the model to learn the series information. Then the Gate Recurrent Unit (GRU) method ^14^ simplifies the LSTM by merging the input and forget gates as update gates. It simplifies the structure but also reduces the ability to express the complexity of the model. Convolutional Neural Network (CNN) ^15^ is mostly used in the field of image processing but it can also be applied to RUL prediction using a one-dimensional structure. Nonetheless, these methods have inherent sequential properties, which hinder the parallelization of training samples.

With the publication of "Attention is all you need" ^16^, the attention mechanism has been gradually applied to time series, inspired by the human visual attention mechanism. It relies on attention to model the whole series without considering the specific position in the sequence, so in this way, parallel computation can be carried out. The attention mechanism takes different weighting factors to make sure the input sequence gets different attention assignments. The Transformer model uses encoder-decoder architecture. In recent years it has been used more and more for time series prediction in the industry. Ding et al. ^17^ designed a new tokenizer and encoder module to extract features from the time–frequency of vibration data and then used Transformer to diagnose the faults. Alexakos^18^ proposed an image classification transformer used to diagnose the vibration images after a short time Fourier transform. Unfortunately, Transformer has not been well mined in the RUL prediction field ^17^, so the advantages of Transformer in avoiding recursion, parallel computation, and reducing performance degradation are not well utilized in RUL prediction.

Based on the above problems, a PSR-former model is proposed using the strong ability of the Transformer in global sequence modeling. The PSR layer further deepens the understanding of vibration features after feature fusion since the features are easily polluted by noise and it is difficult to form HI for prediction. The Transformer layer receives the enhanced vibration features as input to predict the RUL. Bearings are the most important and easily damaged part of rotating machines. Therefore, the bearings are analyzed as an example, and the RMSE value is used as a measurement tool to show the RUL accuracy to illustrate the implementation effect that has achieved 1.0311. Some state-of-art methods are also adopted on the same dataset to compare the result with PSR-former. However, PSR-former still shows good performance after the comparison.

The rest of the paper is organized as follows. “Proposed method” describes the method specifically. “Dataset” introduces the IMS bearing dataset. “Experiment results” processes the dataset using the proposed method and then compares the results with some deep learning algorithms and traditional machine learning methods. “Conclusions” summarizes the main contribution and looks forward to future work.

## Proposed method

**Figure 1:**
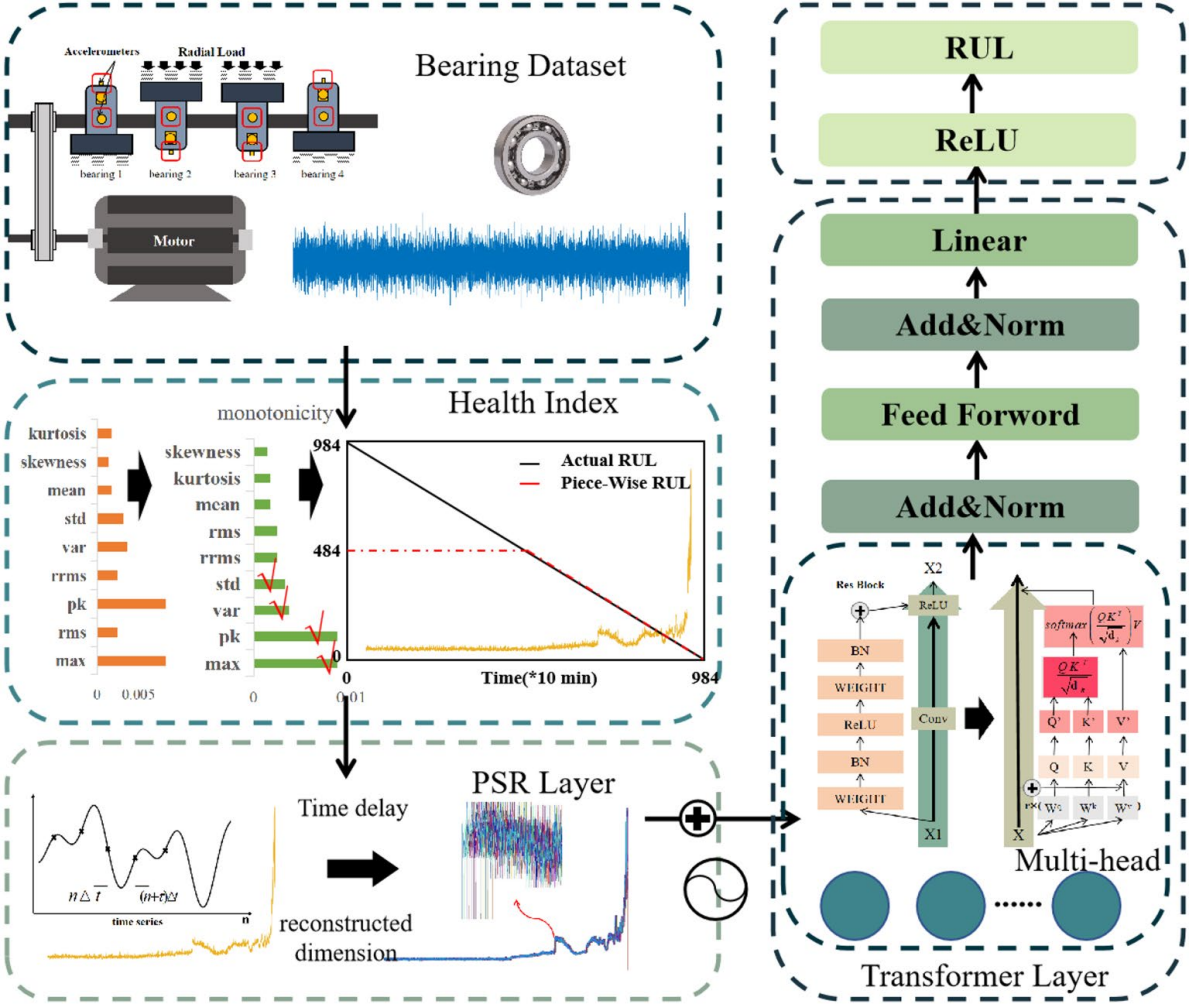
Flowchart of RUL prediction process.

This section describes the PSR-former method, including two important components: the construction of the health index(HI) ^19^ which reflects the health status of equipment in the form of values, and the construction of the PSR-former model. The model was conducted in Python3.8 with PyTorch. The graphics card model is NVIDIA GeForce RTX 2060. The flowchart of the RUL prediction process is shown in Fig. 1. Features of vibration data were extracted and the monotonicity index was chosen to select the features to form a new health index enhanced initially. Then it was input into the proposed PSR-former model to predict the RUL of the bearing. The specific description of each step is as follows.

### A. Construction of the health index

Feature extraction is crucial in RUL prediction ^20^. In general, feature extraction is mainly analyzed from the time and frequency domain perspectives. The time domain analysis method is to analyze and discriminates the signals directly by analyzing the time scale parameters of the signals. It commonly includes statistical features such as mean, variance, kurtosis, deviation, and pulse indicators of the waveform.

The dimensionless time-domain feature is sensitive to the impact energy, but in the case of rolling bearings that wear slowly over time, there is a greater need for a feature that can describe the entire process of degradation.

The sensitivity, regularity, and monotonicity of the bearing fault differ from each other, it is difficult to quantify the degree of performance degradation. Based on this, this paper uses monotonicity ^21^ as the discriminatory criterion for feature selection and accordingly considers that the selection of features should be as clear as possible to reflect the general trend of degradation.

1 $$ Monotonicity(f_{i} ) = \left| {\frac{{Num\, (diff(f_{i} ){ > }0)\,  }}{n - 1} - \frac{{Num\,  (diff(f_{i} ){ < }0)}}{n - 1}} \right| $$

where *n* is the number of sampled signals, $f_{i}$ is the ith characteristic of signals, and $diff(f_{i} )$ is the difference of the *ith* feature of signals.

The features selected in this way can characterize the monotonicity trend of degradation. To better coalesce the features, PCA is used to reconstruct the features that meet the monotonicity discriminant to form new features as the health index of bearing deterioration.

### B. PSR-former model

The most important part attention mechanism in Transformer was first proposed when using cosine functions to measure the similarity between reading and writing keyword vectors and working memory thus allocating different attention. The Transformer completely discards the RNN and CNN architectures and utilizes the attention mechanism to obtain powerful feature extraction and long-distance feature capture capabilities. Based on this, Transformer is increasingly used in temporal processing. However, Transformer is rarely applied to RUL prediction in the industry.

**Figure 2:**
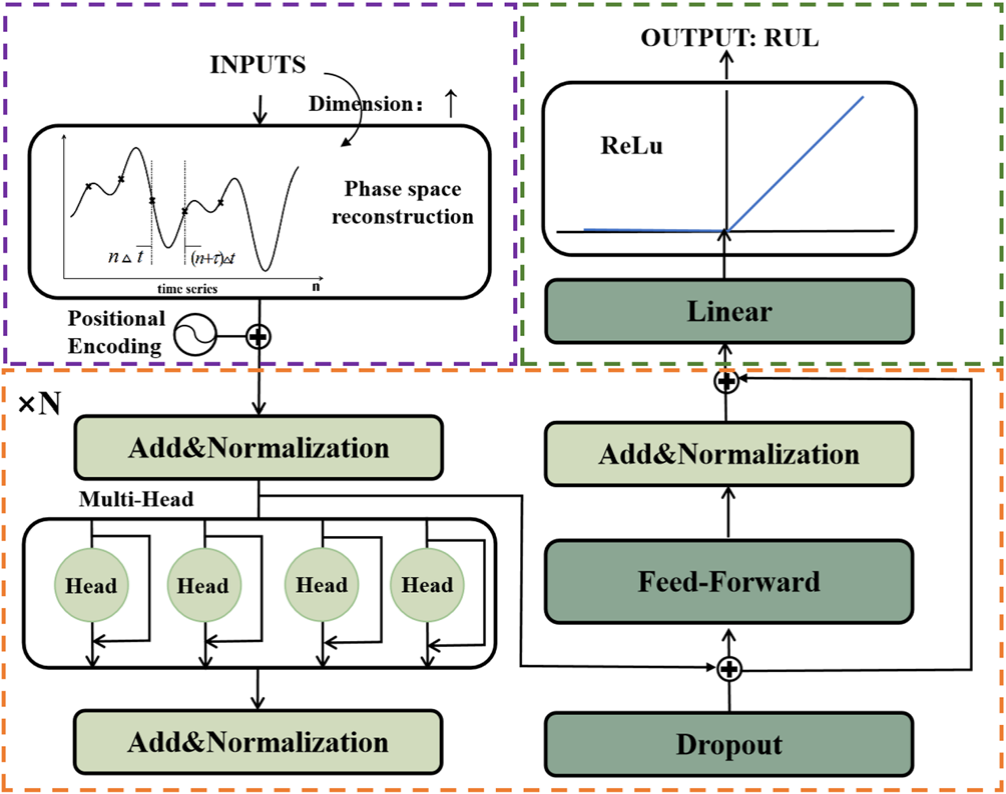
PSR-former model structure.

A PSR-former model based on the Transformer structure was then proposed. The PSR layer enhanced the ability to capture feature information by using the PSR layer in the encoder as the embedding to perform the dimension upgrading operation on the input. The self-attention structure in the encoder is connected as the residual block to enhance the weight of the feature matrix. The decoder layer is replaced by the linear layer to realize the regression problem. The model structure is shown in Fig. 2, where $\tau$ represents the delay time and N means having N identical structures. The purple part in Fig. 2 represents the PSR layer which is also the embedding part of the PSR-former model. The orange part in Fig. 2 represents the main structure of the Transformer structure. Features are further extracted and enhanced by the orange part. The model outputs features as RUL values through a linear layer in the green part of the PSR-former model. The key components of the model are described as follows:

### B.1 PSR feature enhancing layer

In actual industry, the operation of equipment is a complex system. Although the operation of bearings is relatively simple, they can still be easily affected by the surrounding environment. The actual vibration data generated contains a large number of noise and variables making the time series chaotic. Phase Space Reconstruction is the key step of this system ^22^. To further extract the useful information in vibration data, the phase space reconstruction technique was used to map the low-dimensional time series to higher dimensions and thus further find the characteristic laws embedded within the system. In order to realize the mapping from low dimensional space to high dimensional space, the Takens theorem ^23^ was proposed to ensure that the phase space can be reconstructed from the one-dimensional time series in the same sense as the topological equivalence of the original dynamical system. The reconstruction of phase space is realized by coordinate delay. It needs two key parameters, dimension *m* and delay time $\tau$. The common method to determine the delay time $\tau$ is mainly by the mutual information and autocorrelation function method. However, the autocorrelation function can only extract the linear correlation between time series, it is difficult to extend the time delay to higher dimensions. Therefore, the mutual information method is chosen in this paper. Firstly, the maximum time delay *t* for computing the mutual information is selected to obtain the time system *M:*$(m_{1} ,m_{2} ,...,m_{t} )$ and *N:* $(n_{1} ,n_{2} ,...,n_{t} )$ under different time delays where $n_{i} = m_{i} + \tau$.The entropies of information obtained from the two time systems are ^23^:

2 $$ \begin{gathered} H(M) = - \sum\limits_{i = 1}^{t} {P_{i} (m_{i} )} \log {}_{2}P_{m} (m_{i} ) \, \hfill \\ \, \hfill \\ \end{gathered} $$

3 $$ H(N) = - \sum\limits_{i = 1}^{t} {P_{i} (n_{i} )} \log {}_{2}P_{n} (n_{i} ) \, $$

The probability of the occurrence of events $m_{i} ,n_{i}$ is constructed as a boundary statistic in terms of the most value of the sequence. The mutual information of system *N* is obtained by the time system *M*.

4 $$ I(N,M) = H(M) - H(M|N) $$

5 $$ I(N,M) = \sum\limits_{i} {\sum\limits_{j} {P_{mn} (m_{i} ,n_{i} )\log_{2} } } \left[ {\frac{{P_{mn} (m_{i} ,n_{i} )}}{{P_{m} (m_{i} )P_{n} (n_{i} )}}} \right] $$

where:

6 $$ H(N|m_{i} ) = - \sum\limits_{j} {\left[ {P_{mn} (m_{i} ,n_{i} )/P_{m} (m_{i} )} \right]} \log \left[ {P_{mn} (m_{i} ,n_{i} )/P_{m} (m_{i} )} \right] $$

The first minimal value $I(N,M)$ calculated at different time delays is used as the optimal delay time $\tau$ which represents the greatest degree of uncorrelation between the reconstructed time series.

After determining the delay time $\tau$, the dimension *m* is determined by Cao’s method ^24^. A maximum dimension *M* is first determined and then the phase space is reconstructed using the delay time determined before.

$$ \left\{ \begin{gathered} S(1) = [x(1),x(1 + \tau ),...,x(1 + (m - 1)\tau )]\quad  \quad \quad\quad\quad \quad \quad\quad\quad  \quad \quad \quad  { (7)} \hfill \\ S(2) = [x(2),x(2 + \tau ),...,x(2 + (m - 1)\tau )]\quad\quad\quad\quad\quad \quad \quad\quad\quad \quad\quad \quad  { (8)} \hfill \\ ...... \hfill \\ S(k) = [x(k),x(k + \tau ),...,x(k + (m - 1)\tau )] \quad \quad \quad\quad\quad \quad \quad\quad\quad\quad\quad \quad { (9)} \hfill \\ \end{gathered} \right. $$

For each dimension, the distance between the *ith* vector and other vectors is calculated, and for each phase vector $x(i)$, there is a nearest proximal point $x^{N} (i)$. The distance between them is:

10 $$ R_{d} (i) = \left\| {x(i) - x^{N} (i)} \right\| $$

When the dimension of the phase space increases by one dimension, the distance between the phase points will change.

11 $$ R_{d + 1}^{2} (i) = R_{d}^{2} (i) + \left\| {x(i + \tau d) - x^{N} (i + \tau d)} \right\| $$

If $R_{d + 1} (i)$ is much larger than $R_{d} (i)$, then consider the adventitious point false. Define *a* in the following.

12 $$ a(i,d) = \frac{{R_{d + 1}^{2} (i) - R_{d}^{2} (i)}}{{R_{d} (i)}} = \frac{{\left\| {x(i + \tau d) - x^{N} (i + \tau d)} \right\|}}{{R_{d} (i)}} $$

then

13 $$ a(i,d) = \frac{{R_{d + 1}^{2} (i) - R_{d}^{2} (i)}}{{R_{d} (i)}} = \frac{{\left\| {x(i + \tau d) - x^{N} (i + \tau d)} \right\|}}{{R_{d} (i)}} = \frac{{\left\| {x(i + \tau d) - x^{N} (i + \tau d)} \right\|}}{{\left\| {x(i) - x^{N} (i)} \right\|}} $$

So $a(i,d)$ can be:

14 $$ a(i,d) = \frac{{\left\| {x_{d + 1} (i) - x_{d + 1}^{N} (i)} \right\|}}{{\left\| {x_{d} (i) - x_{d}^{N} (i)} \right\|}} $$

$x_{d} (i)$ and $x_{d}^{N} (i)$ are the *ith* vector in the d-dimensional space and its most proximal point. By calculating $E_{1} (m)$ and $E_{2} (m)$, where $E_{2} (m)$ is a judgment criterion defined to better observe the change of $E_{1} (m)$, the embedding dimension *m* can be judged when $E_{1} (m)$ and $E_{2} (m)$ are constant.

15 $$ E_{i} (m) = E_{i}^{*} (m + 1)/E_{i}^{*} (m) $$

where:

16 $$ E_{1}^{*} (m) = \frac{1}{N - m\tau }\sum\limits_{i = 1}^{N - m\tau } {a(i,m)} $$

17 $$ E_{2}^{*} (m) = \frac{1}{N - m\tau }\sum\limits_{i = 1}^{N - m\tau } {\left| {x(i + m\tau ) - x^{N} (i + m\tau )} \right|} $$

When $E_{1} (m)$ is essentially stable, the dimension *m* at this point is the embedding dimension needed for the reconstruction. Similarly, the dimension *m* selected ensures the minimum correlation between sequences. The definition of $E_{2} (m)$ is based on the uncorrelation sequences. It always equals 1.

After getting the reconstructed dimensions to reconstruct the time series, Transformer uses the sliding window to divide the reconstructed time series data to get a sequence of time window data blocks. The data shape of each sample is: $[num,time,feature]$, where *num* is the number of time window sequences, *time* is the length of the time window, and *feature* is the number of reconstructed dimensions. The Transformer uses the global information but not the sequential information of the sequence. It needs to calculate the relative position of each sequence using position embedding. Its calculation formulas are:

18 $$ \begin{gathered} PE(pos,2i) = \sin (pos/10000^{\frac{2i}{d}} ) \, \hfill \\ \, \hfill \\ \end{gathered} $$

19 $$ PE(pos,2i + 1) = \cos (pos/10000^{\frac{2i}{d}} ) \, $$

where *d* is the dimension of the sequence. The results of the sequence after feature enhancement and the result after position embedding are added to obtain the representation vector of the sequence as the input to the model.

### B.2 Multi-head attention

The self-attention structure requires the query vector *Q*, the key vector *K* and the value vector *V. Q, K,* and *V* are obtained by multiplying the input matrix *X* by the matrix $W^{Q} ,W^{K} ,W^{V}$, where $W^{Q} ,W^{K} ,W^{V}$ are trainable projection matrices. The multi-head attention layer uses the time series data to do the dot calculation. The output obtained is as follows:

20 $$ Attention(Q,K,V) = soft\max (\frac{{QK^{T} }}{{\sqrt {d_{k} } }})V $$

**Figure 3:**
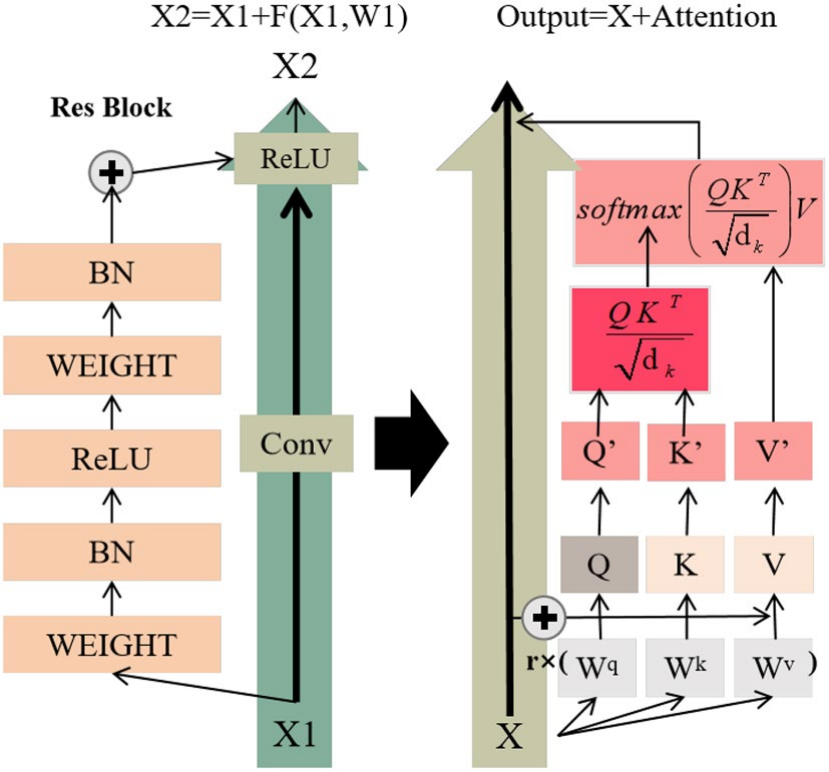
Layer-hopping connection mode.

Inspired by the residual network, the residual unit can solve the degradation problem of the network and make convergence faster by connecting the input and attention fractions through layer hopping. Cao et al. ^25^ showed that the residual connection is an effective way to train the network to transfer information across layers and prevent the gradient from disappearing and exploding. Based on this, a layer-hopping was also performed in the calculation of *Q, K, V* so that the original *Q, K, V* becomes *Q', K', V'*. The structure is shown in Fig. 3. The projection matrix $W^{Q} ,W^{K} ,W^{V}$ are multiplied twice by the coefficient *r* and the input matrix *X* to obtain *Q, K* and *V*, and then they are connected with *X* by layer hopping.

21 $$ \left\{ \begin{gathered} Q = X \times rW^{q} \hfill \\ K = X \times rW^{k} \hfill \\ V = X \times rW^{v} \hfill \\ \end{gathered} \right. $$

22 $$ \left\{ \begin{gathered} Q^{{\prime}} = X + X \times rW^{q} \hfill \\ K^{{\prime}} = X + X \times rW^{k} \hfill \\ V^{{\prime}} = X + X \times rW^{v} \hfill \\ \end{gathered} \right. $$

The transformation matrix is multiplied by the coefficient *r*. The query vector *Q* is used as the analysis to show the loss back-propagation. The computation without taking a jump layer connection is:

23 $$ \frac{\partial L}{{\partial X}} = \frac{\partial L}{{\partial Q}} \cdot \frac{\partial Q}{{\partial X}} = W^{q} \frac{\partial L}{{\partial Q}} $$

And when the idea of residuals is used for layer-hopping connections, the loss is.

24 $$ \frac{\partial L}{{\partial X}} = \frac{\partial L}{{\partial Q^{{\prime}} }} \cdot \frac{{\partial Q^{{\prime}} }}{\partial X} = (1 + rW^{q} )\frac{\partial L}{{\partial Q^{{\prime}} }} $$

It can be known that when this connection is used, the matrix has a greater range of variation during propagation and it is updated faster during gradient descent. Similarly, the Attention score is computed and then connected with the input *X* hopping layer to obtain *X* + *Attention*.

Each Attention value result is computed as a Head, and multiple Heads are copied and spliced together in the Multi-Head layer.

$$ MultiHead(Q^{\prime},K^{\prime},V^{\prime}) = Concat(head_{1} ,head_{2} ,...,head_{h} )W^{O} $$

where $W^{O} \in R^{{d_{{{\text{model}}}} *d_{{{\text{model}}}} }}$, $d_{{{\text{model}}}}$ is the input dimension of the sequence.

After Multi-Head Attention, the sequences need to go through the Add&Norm layer which consist of an add layer and a normalization layer. The add layer also takes a residual connection to make the network focus on the current difference part. The norm layer makes the output of each layer gets normalized to speed up the convergence.

$$ \begin{gathered} LayerNorm(X + MultiHeadAttention(X)) \hfill \\ LayerNorm(X + FeedForward(X)) \hfill \\ \end{gathered} $$

## Dataset

**Figure 4:**
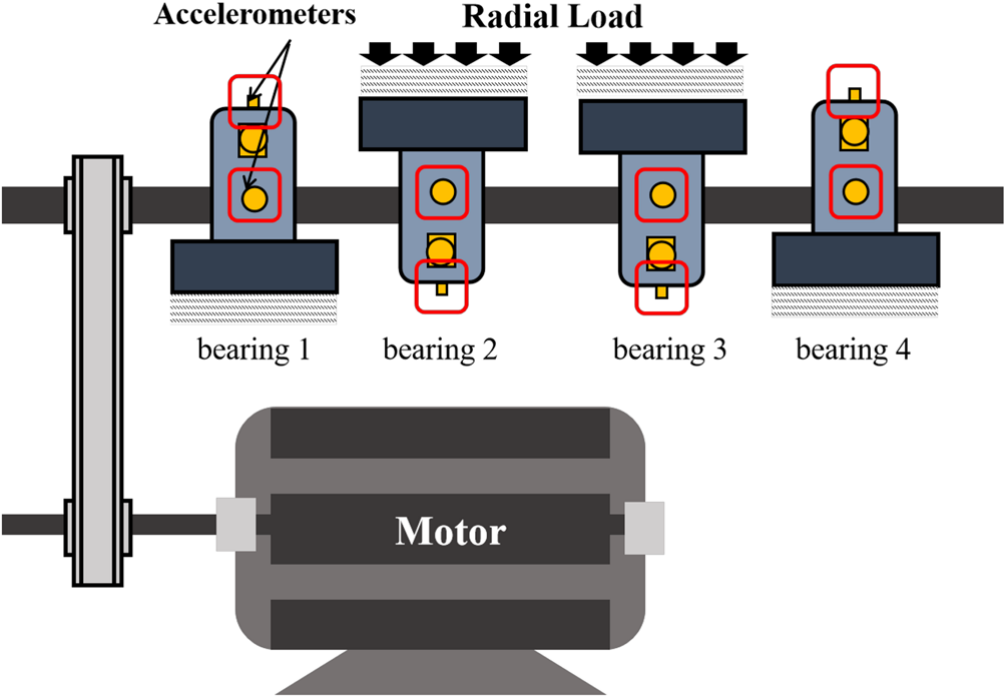
Installation diagram of bearings and sensors on IMS.

The data was provided by the Center for Intelligent Maintenance Systems (IMS), University of Cincinnati ^26^. The installation of bearings and sensors is shown in Fig. 4. The test rig is mainly composed of a motor, belt, shaft, bearing, sensors, and additional radial load. The sensors are high sensitivity PCB 253B33 QuartICP accelerometer located on the bearing. It contains three datasets, representing the whole process of bearing from normal operation to failure. There are four bearings in each dataset. The bearing type is Rexnord ZA-2115. The shaft speed of the test rig is 2000 rpm and the radial load is 6000 lbs. The sampling frequency is 20.48 kHz, the sampling time is 1 s, and the period is 10 min. The endurance duration of the three datasets is 828 h, 164 h, and 741.3 h. The fault location appeared in the inner ring, rolling element, and outer ring respectively in the different datasets which are specifically described in Table 1. The oil return pipe lubricated with oil is provided with a magnetic plug. When the debris adsorbed on the magnetic plug reaches the threshold value, it is considered that the bearing has been completely degraded, then the collection work stops. A description of the three different datasets is shown in Table 1.

**Table 1:** Description of three datasets.

	Num of DOC	Num of channel	Duration	Fault location
Dataset 1	2156	8	828 h	Bearing 3: inner ring Bearing 4: rolling element
Dataset 2	984	4	164 h	Bearing 1: outer ring
Dataset 3	4448	4	741.3 h	Bearing 3: outer ring

## Experiment results

This section provides the results of each step in this paper including feature reconstruction, PSR-former, and comparative experiments. The feature reconstruction step initially enhanced the degradation features of the bearing. PSR-former was the main structure in this paper. It combined the PSR layer with the Transformer layer. Then comparative experiments were conducted on the dataset.

### A. Feature reconstruction

**Figure 5:**
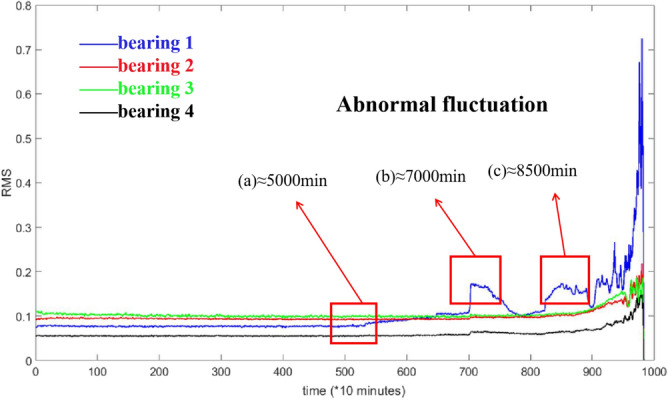
Process of bearing RMS for dataset 2.

Dataset2 was used as an example to illustrate the experiment which describes the whole process of four bearings with only bearing 1 from normal to failure. The outer ring fails at last. The full RMS process of bearing 1 is shown in Fig. 5.

The expression of RMS is:

25 $$ RMS{ = }\sqrt {\frac{1}{N}\sum\limits_{i = 1}^{N} {x_{i}^{2} } } $$

where $x_{i}$ denotes the *ith* point of the data.

It can be seen from Fig. 5 that the whole process of bearing degradation can be approximately divided into four stages according to the change of amplitude. The abscissa in Fig. 5 is the time point of bearing operation and the ordinate is the RMS value of bearing according to the time point. The four stages were roughly determined: (a) At about 5000 min, the curve shows a small upward fluctuation, indicating that the bearing starts to fail here. (b) At about 7000 min, the curve shows a large step, and then it slowly declines with similar fluctuation amplitude after (a) point. (c) At about 8500 min, the RMS shows a large oscillation, indicating that the bearing has reached a serious failure at this time. Therefore, the degradation process of the bearing can be roughly divided into four categories, 0–(a) for normal operation; (a)–(b) for mild failure; (b)–(c) for moderate failure; and (c)–last for severe failure. Hence, it can be considered that after point (a), the bearing began to deteriorate gradually.

**Figure 6:**
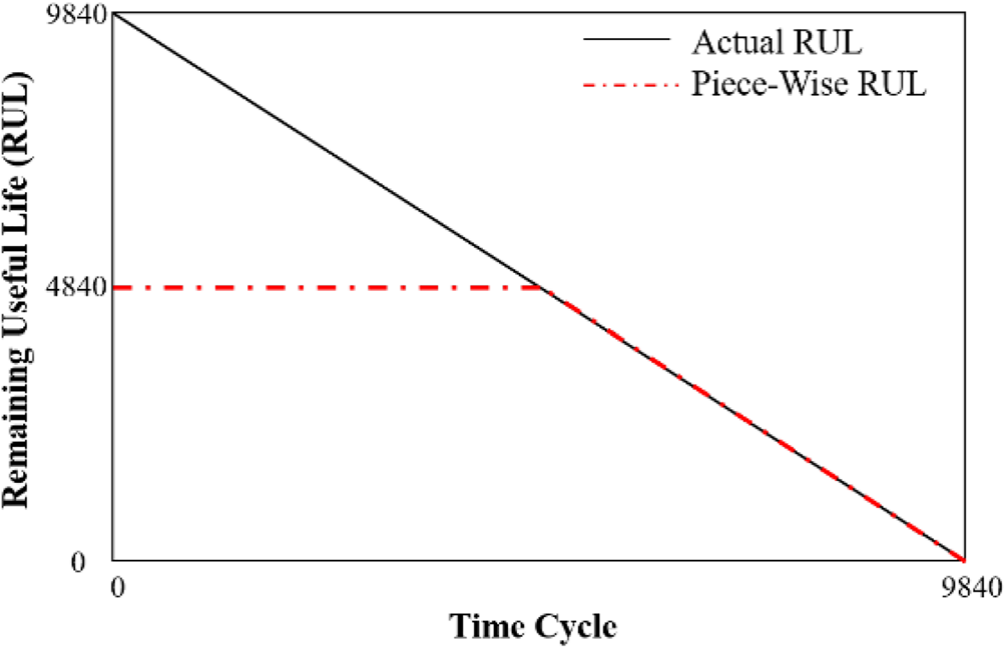
Piece-wise RUL.

In order to better discriminate the RUL of the bearing, the RUL of the data was truncated, and the highest value of RUL was set to 484 × 10 min according to (a) point. i.e., the RUL values of the time cycle from 0 to 5000 min are 484 × 10 min and it is shown in Fig. 6. This operation mapped the HI to the RUL label. The accuracy of artificial division will not affect the judgment of the model on the degradation process since 484 × 10 min is located in the health stage. It did not contain the characteristics of bearing wear.

To show the degradation trend of four bearings in the same dataset, a new feature Reconstructed Root Mean Square (RRMS) was defined based on Root Mean Square (RMS). The definition of RRMS is:

26 $$ RRMS = \frac{RMS}{{RMS_{norm} }},{\kern 1pt} {\kern 1pt} {\kern 1pt} {\kern 1pt} {\kern 1pt} {\kern 1pt} RMS_{norm} = \frac{1}{k}\sum\limits_{i = 1}^{k} {RMS(i)} $$

**Figure 7:**
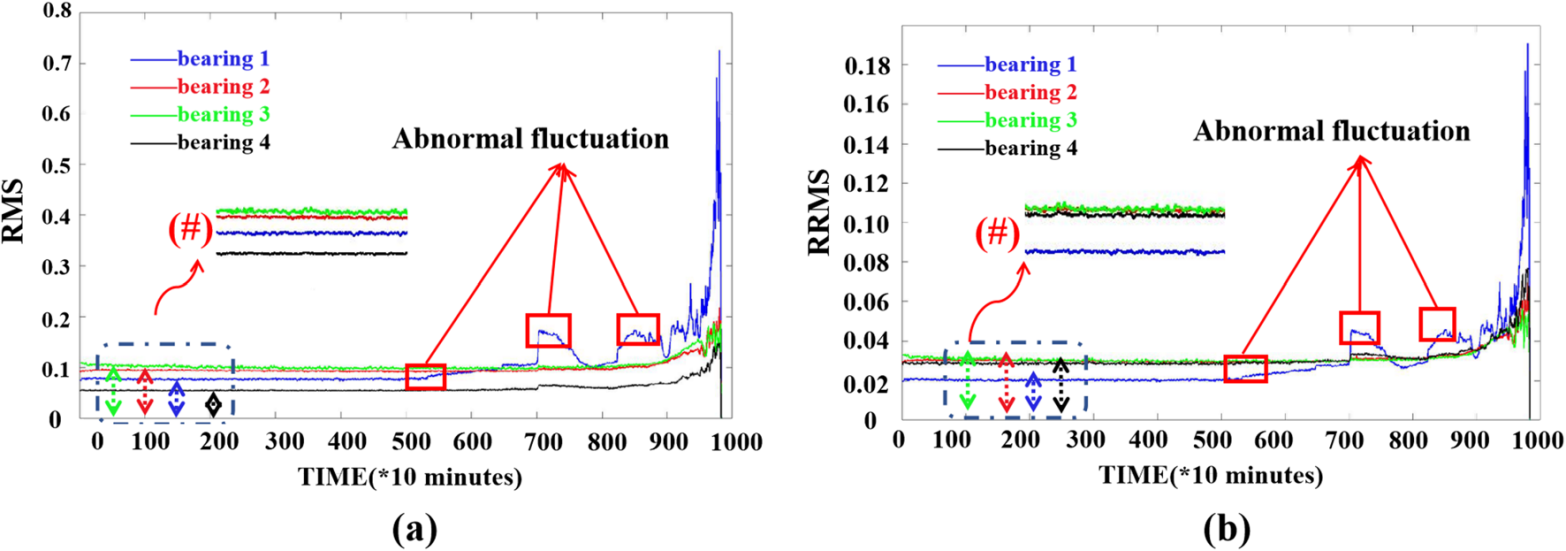
Plots of RMS vs. RRMS (**a**) RMS; (**b**) RRMS.

By comparing the RMS and RRMS of bearings in Fig. 7, it can be seen from (#) in Fig. 7 that RRMS is more beneficial to reduce individual differences. Since there are four bearings in each dataset in IMS bearing data, RRMS can more intuitively distinguish the degradation process of different bearings, although it has the same trend of change as RMS. Hence the RRMS was chosen as one of the features in the selection of RMS and RRMS. The obtained time domain features are shown in Table 2. Where $x$ is the series of vibration data, $N$ is the number of the data point, $\mu$ is the mean value of the whole vibration data and $\sigma$ is the standard deviation.

**Table 2:** Time domain features.

$Mean = \frac{1}{N}\sum\limits_{i = 1}^{N} {x_{i} }$	$Pk = max\{ \left| {x_{i} } \right|\}$	$RMS = \sqrt {\frac{1}{N}\sum\limits_{i = 1}^{N} {x_{i}^{2} } }$
$RRMS(i) = \frac{RMS(i)}{{\frac{1}{N}\sum\limits_{i = 1}^{N} {RMS(i)} }}$	$Max = max\{ x_{i} \}$	$Std = \sqrt {\frac{1}{N}\sum\limits_{i = 1}^{N} {(x_{i} - \mu )^{2} } }$
$Kurtosis = \frac{{E[(x - \mu )^{4} ]}}{{\sigma^{4} }}$	$Skewness = \frac{{E[(x - \mu )^{3} ]}}{{\sigma^{3} }}$	$Var = \frac{1}{N}(\sum\limits_{i = 1}^{N} {(\left| {x_{i} } \right| - \mu )^{2} } )$

**Figure 8:**
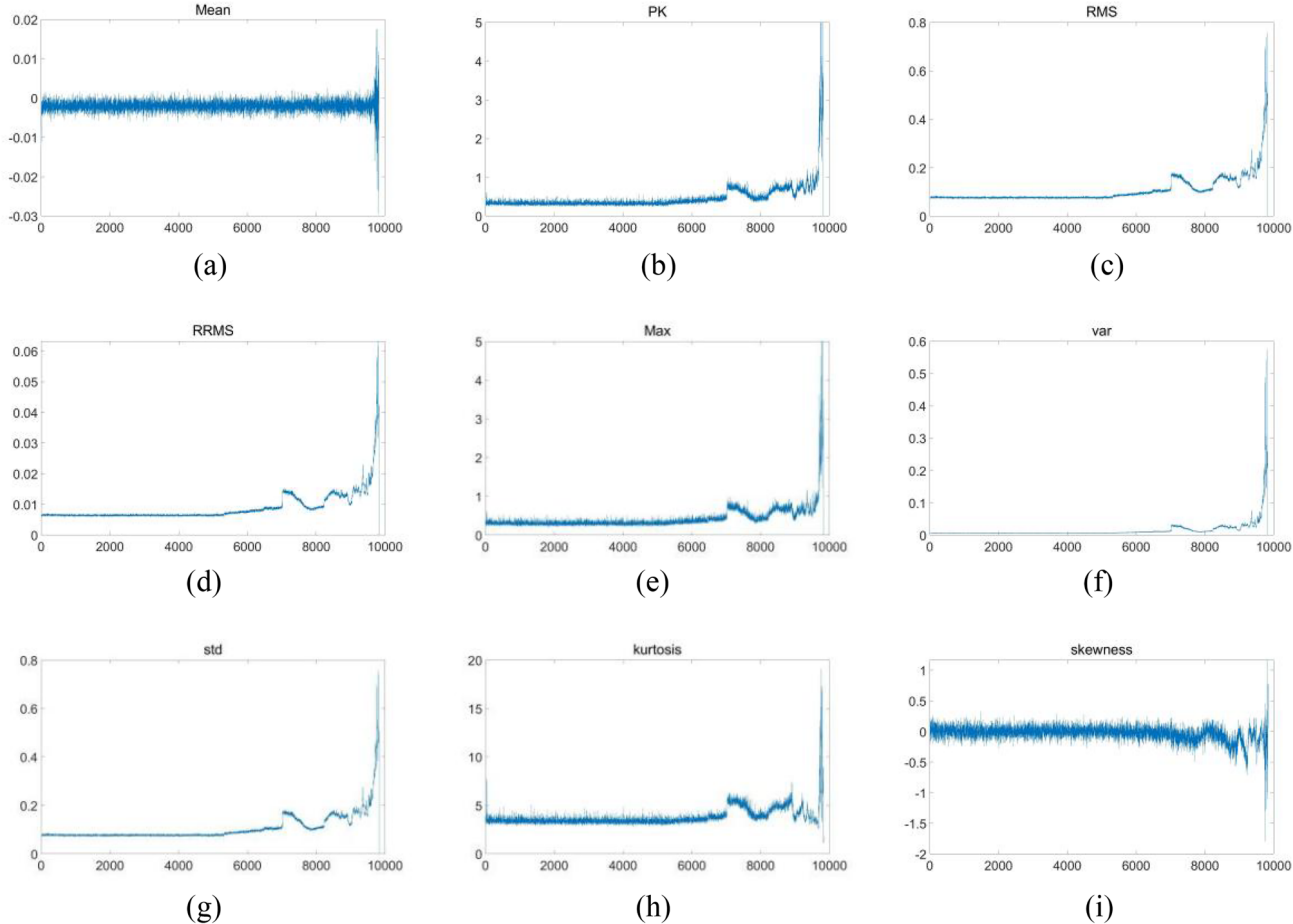
Time-domain features of dataset2 bearing 1 (**a**) mean; (**b**) peak; (**c**) RMS; (**d**) RRMS; (**e**) max; (**f**) var; (**g**) std; (**h**) kurtosis; (**i**) skeweness.

Other features of the full process curve of dataset2 bearing1 from the time domain perspective were extracted in Fig. 8, which shows nine features of bearing in dataset2 including mean, peak value(*PK*), RMS, RRMS, max value, variance(*Var*), standard deviation(*Std*), kurtosis, and skewness.

**Figure 9:**
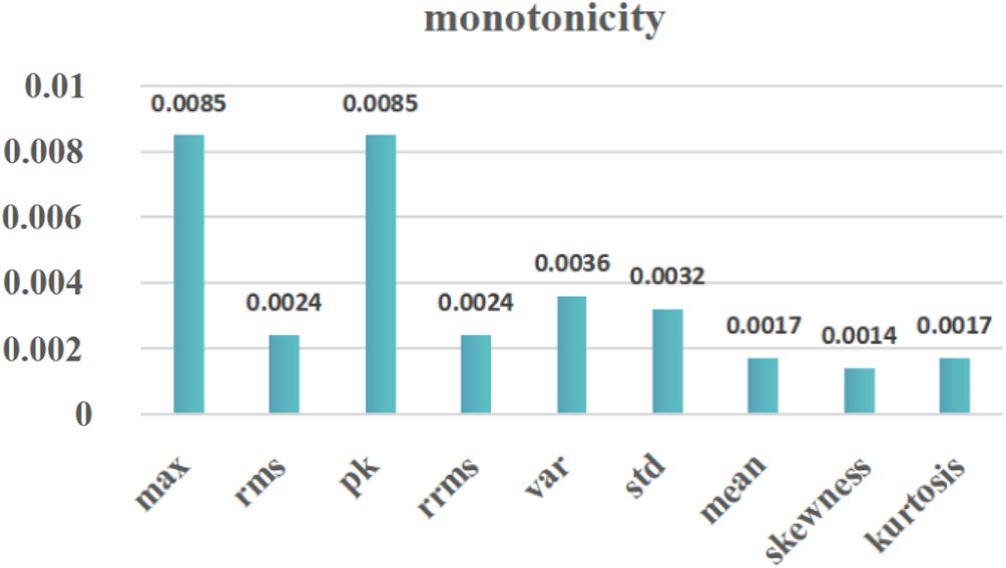
Monotonicity of different features.

The purpose of feature screening is to select clear features that reflect the general trend of degradation as much as possible. The extracted features were selected according to monotonicity. The monotonicity value of each feature is shown in Fig. 9. The top four features were selected. However, the Peak value and the max value have the same trend, and the RMS and the RRMS have the same trend, so one of them was selected. Then *PK, RRMS, Var*, and *Std* were selected as the four features that represent the time series features finally. The feature reconstruction of the four features by PCA found that the first principal component accounted for 96% of the total, thus the new principal component features were reconstructed to obtain.

27 $$ principle1 = 0.5014 \times PK + 0.5031 \times RRMS + 0.4923 \times Var + 0.5031 \times Std $$

After the feature reconstruction step, the features were initially enhanced.

### B. PSR-former

**Figure 10:**
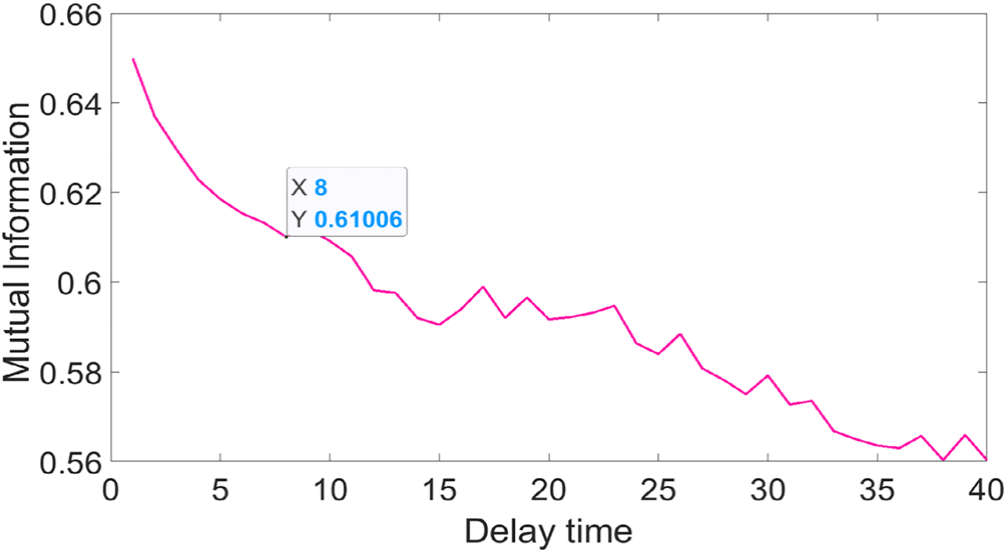
Time delay curve.

The reconstructed time series features are then input into the PSR-former model established to judge the RUL of the bearing. First, the PSR layer of the PSR-former reconstructs the time series. The maximum time delay *t* is determined according to the mutual information method. The curve of mutual information with delay time was plotted to obtain the first minimal value point in Fig. 10. It shows the determined time delay *t* = 8.

**Figure 11:**
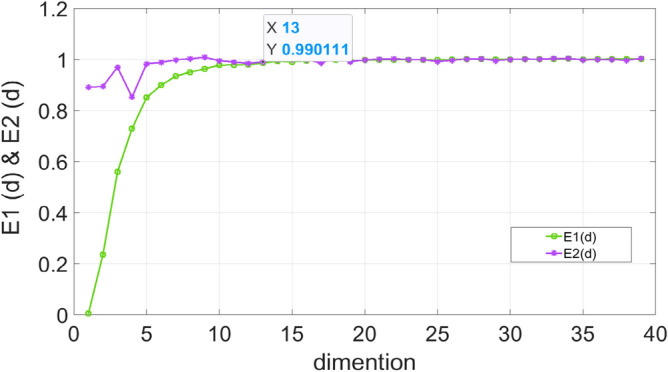
Dimensional determination chart.

After determining the time delay *t*, the phase space was initially reconstructed according to the time delay, and the magnitude of $E_{1} (m)$ and $E_{2} (m)$ values were calculated according to the Cao’s method ^24^, and the variation curves of $E_{1} (m)$ and $E_{2} (m)$ in different dimensions are plotted as shown in the following Fig. 11.

**Figure 12:**
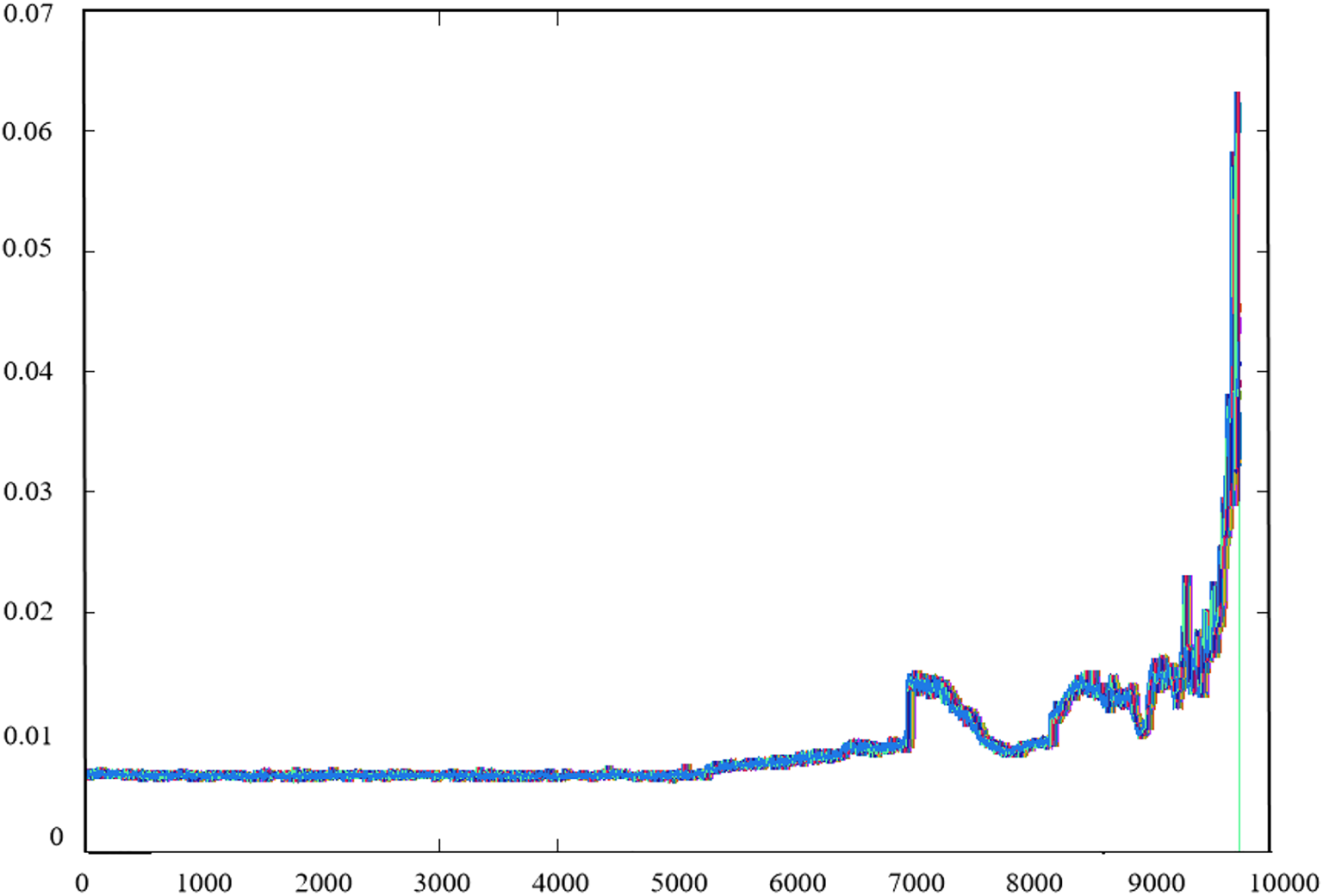
Result after the PSR layer.

**Figure 13:**
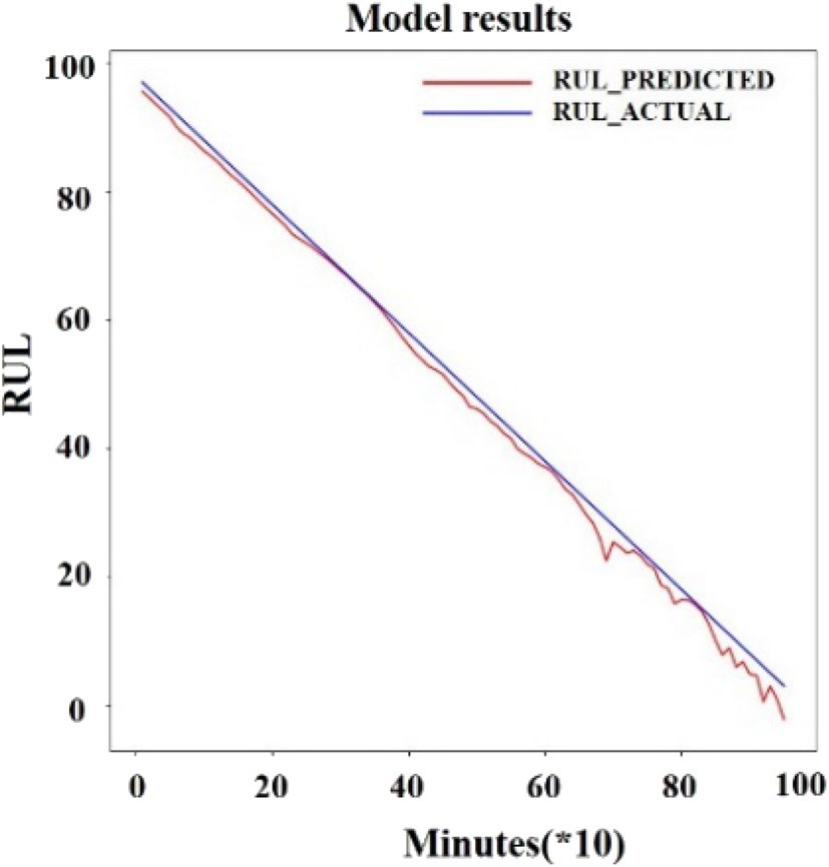
Test result in dataset 2.

Through the curves, it can be observed that when the dimensions $m \ge 13$,$E_{1} (m)$ and $E_{2} (m)$ converge, there is no fluctuation after a certain point. Then the characteristic dimension of the time series was extended to 13 through the PSR layer by Eqs. (7)–(9). The degradation characteristics were enhanced after the PSR layer shown in Fig. 12. The time series after the enhancement is then input into the Transformer, and the parameters of the model were evaluated according to its prediction results for RUL. The highest value of RUL is set to 484 × 10 min by truncation. For the Encoder layer, the dimensionality of encoder *d_model* is set to 14, the total dimension of the time series. The number of layers *N* is 4. For multi-head attention, the *head* is 7 and the jump connection coefficient *r* is 1, the *dropout* is set to 0.4, the optimizer is Adam optimizer and the *learning rate* is 0.001. Set the test data to the last ten percent of the total data to predict the RUL to show the service life of the bearing before the final collapse. The training set and the validation is 4:1. The dimension in the Feed-Forward layer is 64. The batch size is 64, the epoch is 1000 and the step is 1. The dataset2 was trained and tested. To speed up the training process, the input data were normalized so the interval range becomes [−1, 1]. The RMSE of the test set was calculated, and the results of the model were compared by RMSE. The definition of RMSE is as follows:

28 $$ RMSE = \sqrt {\frac{1}{N}\sum\limits_{i = 1}^{n} {(y_{i} - \overline{y}_{i} )^{2} } } $$

where $y_{i}$ is the true RUL value and $\overline{y}_{i}$ is the predicted RUL value. The results obtained from the test set is showed in Fig. 13.

**Figure 14:**
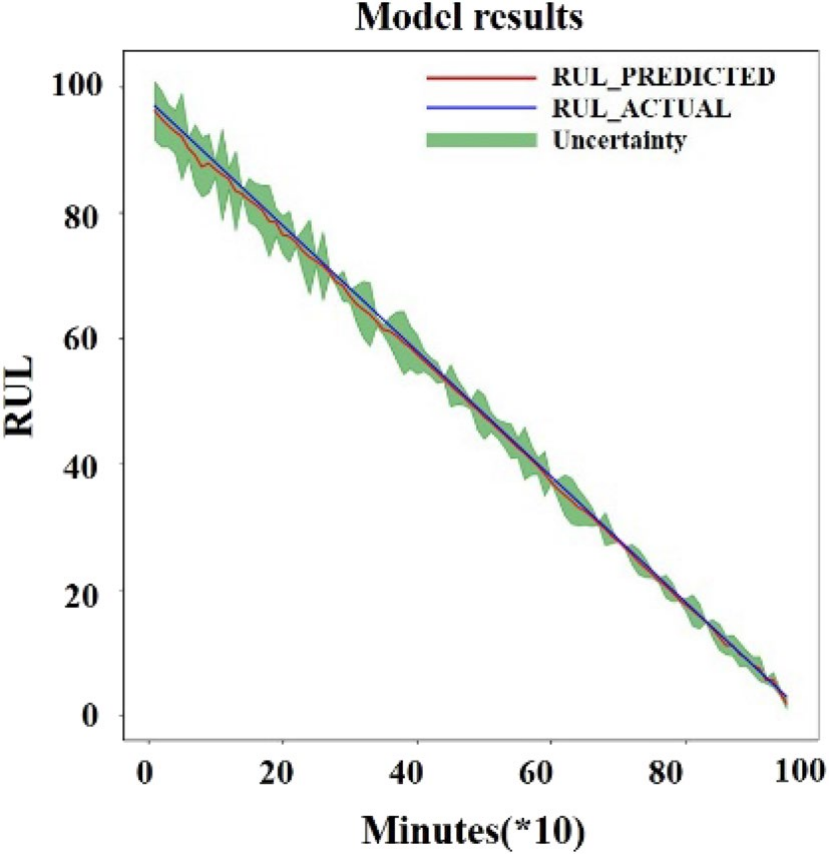
Test result with uncertainty in dataset 2.

The RMSE value obtained from the test set after the normalization of the data is 0.0014. The RMSE value after inverse normalization is 1.0311. To make the prediction more reliable, uncertainty estimation was added to the forecast. Uncertainty estimation combined with deep learning is mainly applied to images to estimate the boundaries. Gal ^27^ divided uncertainty into two main categories, aleatoric uncertainty, and Epistemic uncertainty. Aleatoric uncertainty mainly originates from the data itself. Epistemic uncertainty mainly measures the uncertainty of the estimated parameters of the model during the training process. The model fusion approach ^28^ was adopted to estimate the uncertainty of the model. Use the mean as the predicted value and variance as the uncertainty to do epistemic uncertainty estimation. The results are obtained as shown in Fig. 14.

**Figure 15:**
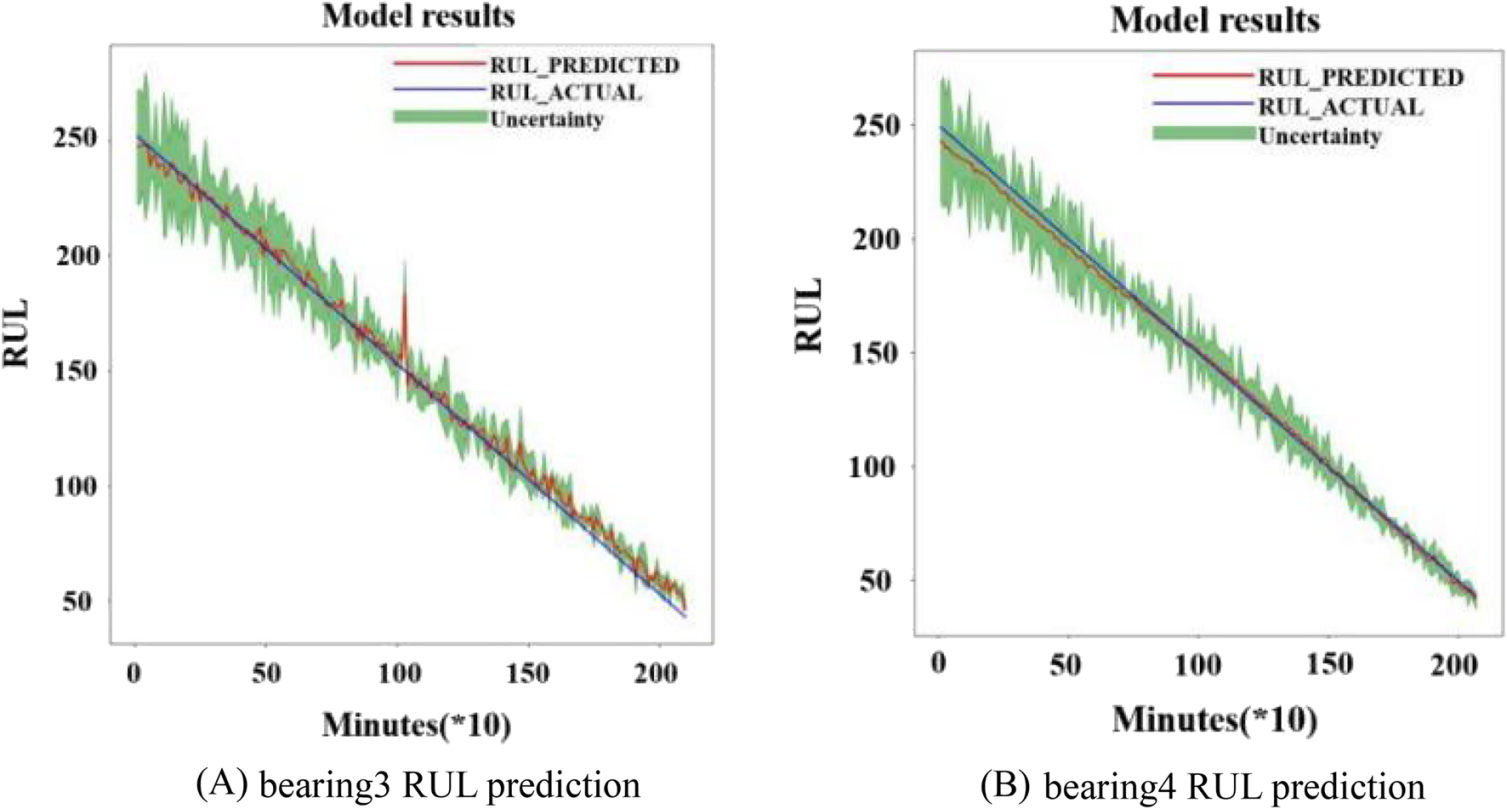
Test result with uncertainty in dataset 1; (**A**) bearing3; (**B**) bearing4.

From the results, it can be seen that the predicted results are accurate to some extent and the uncertainty interval is distributed evenly around the actual RUL. Dataset1 operation is completed with bearing3 having inner ring failure and bearing4 having rolling element failure. The parameters of the PSR are determined with delay time *t* = 2 and dimension *m* = 15 after analysis on bearing3 and bearing4. Then the RUL of the two bearings of dataset1 were predicted based on the same training parameters shown in Fig. 15 where the average RMSE value of bearing3 is 3.7641 and the average RMSE value of bearing4 is 1.9729.

The prediction for bearing3 fluctuates more than bearing4 in Fig. 15. There are more unusual fluctuations in the middle of the test set and the predicted value is slightly larger than the accurate value in the second half of the test set of bearing3. The prediction for bearing4 shows that the predicted value is slightly smaller than the accurate value in the first half of the test set. Overall, the predictions are accurate to some extent.

### C. Comparative experiments

TO verify the effectiveness of the method, comparative experiments were conducted to compare the results of the PSR-former with and without the PSR layer and the results of the analysis of dataset 2 using different methods, respectively. The data used in comparative experiments are data in dataset2 without the upgrade dimension process. At present, the state-of-art methods in RUL prediction are mainly based on machine learning. In order to compare the methods in this paper with those adopted in the current field, the algorithms are classified into two categories: the method based on deep learning and the method based on traditional machine learning. In deep learning methods, Transformer^29^, RNN^30^, LSTM^31^, GRU^32^, 1D-CNN^33^, and CNN combined with LSTM^34^ were chosen under the same data settings as proposed method in this paper to analyze and they were all connected in three layers whose hidden dimension is 14 and 64 to be as close as possible to the PSR-former model. The other parameters of the networks such as batch size and learning rate were adjusted to the best. Random Forest(RF)^35^, Support Vector Machine(SVM)^36^, Linear Regression(LR)^37^, Logistic Regression(LOR)^38^, and K-Nearest Neighbours(KN)^39^ were chosen as traditional machine learning algorithms to analyze the data. In addition, Stacked-autoencoder-LSTM(SAE-LSTM) model^40^ and Bidirectional (BiLSTM) model^41^ in the class of deep learning were tested through similar data in this paper. The two models were trained according to the literature which were built on the same IMS bearing dataset. The SAE-LSTM model structure was 5–20–5 which is the size of different hidden layers of SAE-LSTM. However, the parameters in the BiLSTM were not fully recorded, to maintain consistency, the unrecorded parameters are the same as the SAE-LSTM and the model structure was also 5–20–5. Then the two models were tested using the same test data as PSR-former model.

**Figure 16:**
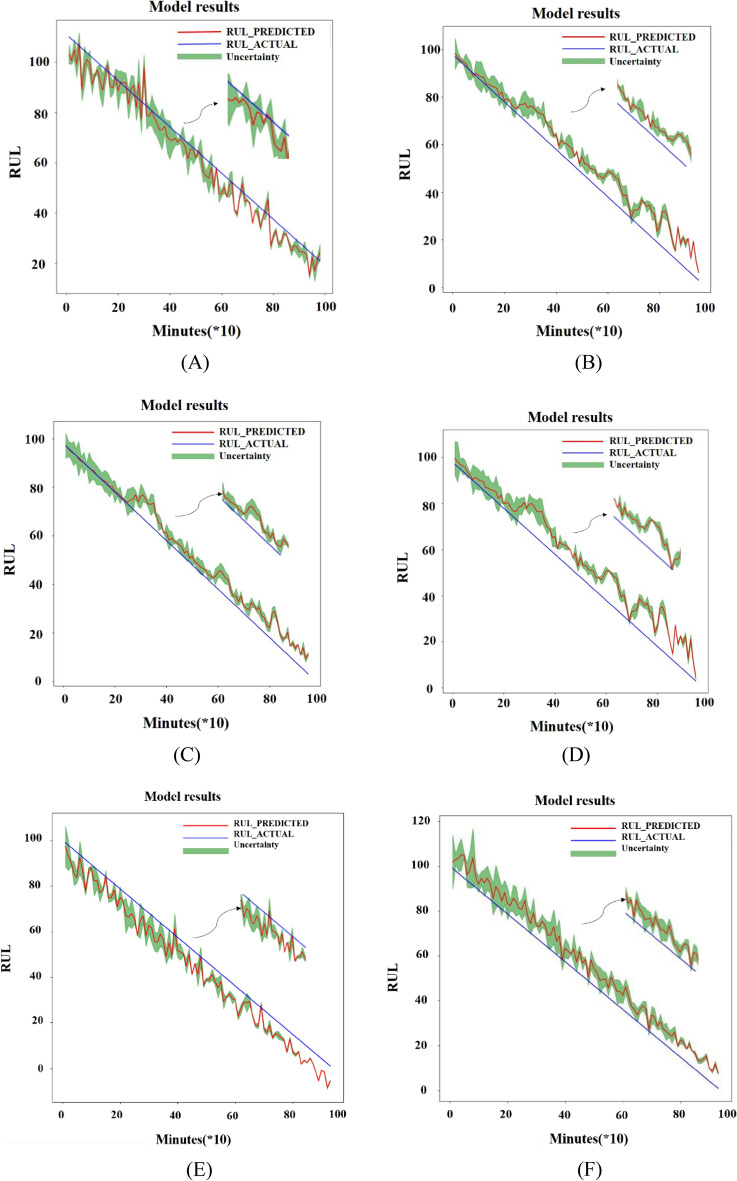

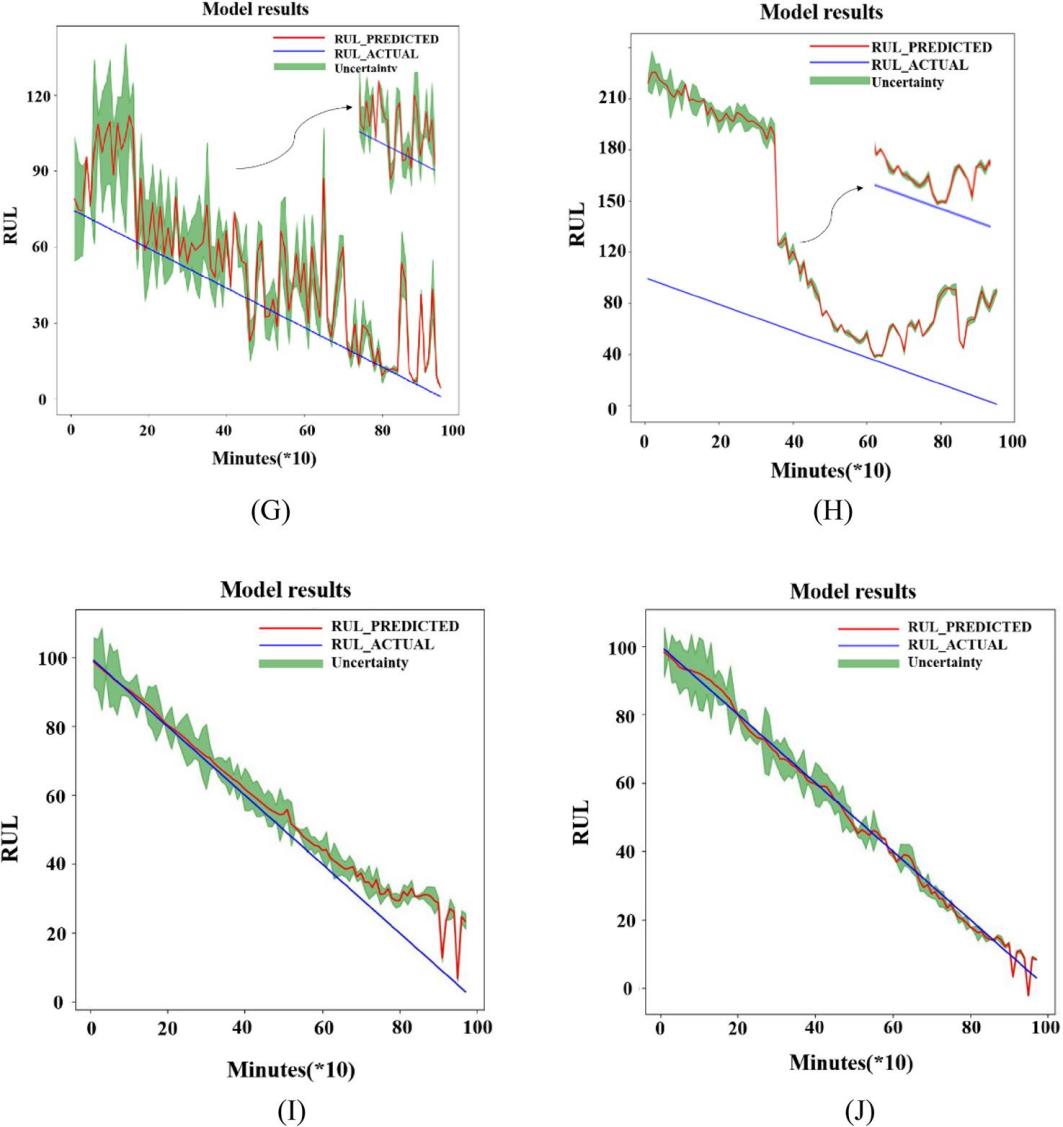
Visualization of RUL prediction results of different comparison algorithms. (**A**) Transformer; (**B**) RNN; (**C**) LSTM; (**D**) GRU; (**E**) 1-D CNN; (**F**) CNN-LSTM; (**G**) RF; (**H**) SVM; (**I**) SAE-LSTM; (**J**) BiLSTM.

**Figure 17:**
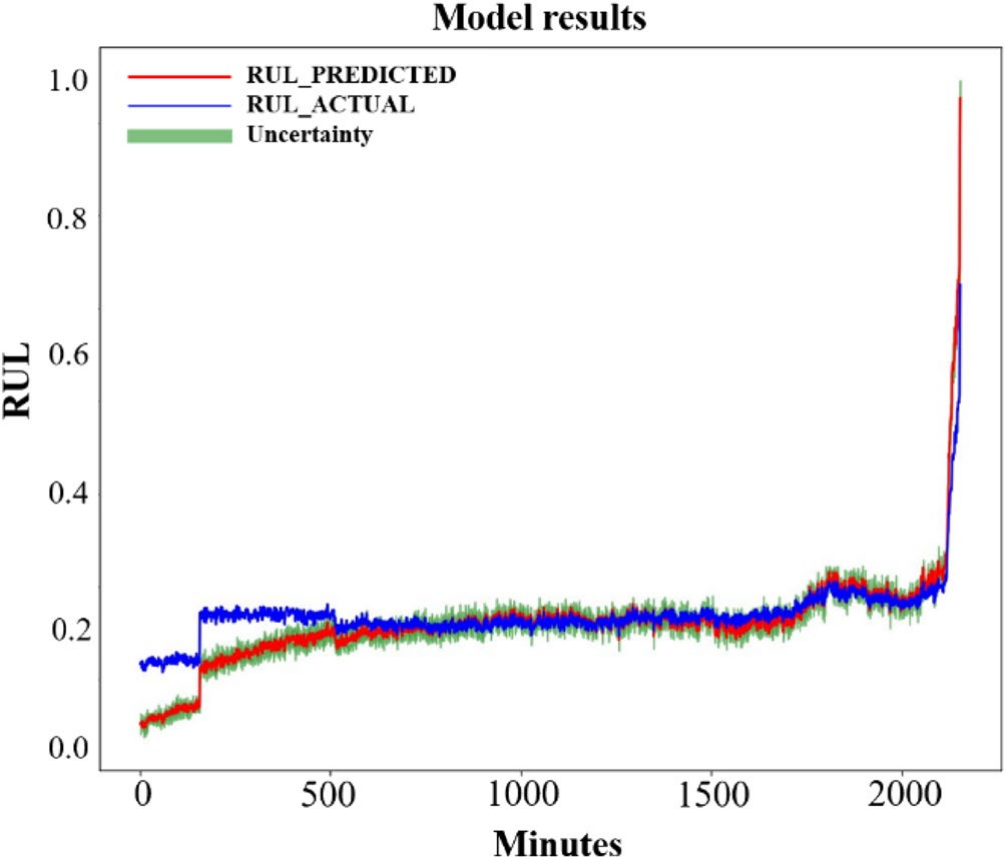
Visualization of retest results using bearing3 data.

If the predicted RUL deviates greatly from the actual RUL, the RMSE value will be too large. At this time, the prediction result is not ideal, which is meaningless for analysis. Therefore, a threshold is chosen for RMSE which is set to 100, then the predicted result of RMSE less than 100 is plotted in Fig. 16. In Fig. 16, (A) is the result of Transformer, (B) is the result of RNN, (C) is the result of LSTM, (D) is the result of GRU, (E) is the result of one-dimensional CNN (1D-CNN), (F) is the result of CNN-LSTM, the combination of CNN and LSTM, (G) is the result of RF, (H) is the result of SVM, (I) is the result of SAE-LSTM, and (J) is the result of BiLSTM. The blue line in the graph represents the real RUL of the bearing, the red line is the predicted RUL, and the green interval represents the uncertainty of the prediction results. The interval obtained from multiple predictions can make the errors between real and predicted RUL more intuitive. The specific values of RMSE results are recorded in Table 3. In Table 3, (1) is Transformer, (2) is RNN, (3) is LSTM, (4) is GRU, (5) is 1D-CNN, (6) is CNN-LSTM, (7) is SAE-LSTM, (8) is BiLSTM, (9) is RF, (10) is SVM, (11) is LR, (12) is LOR, (13) is KN, and the last one is the method proposed in this paper. In addition the trained model was also validated through bearing3^41^. The last 1/3 prediction result is shown in Fig. 17. From the result, it can be seen that the model can accurately predict the final stage of bearing failure.

**Table 3:** RMSE results of different methods under the same data settings.

Methods	(1) Transformer	(2) RNN	(3) LSTM	(4) GRU
Deep learning	6.9779	9.8762	7.1399	10.1544
	(5) 1D-CNN	(6) CNN-LSTM	(7) SAE-LSTM	(8) BiLSTM
	8.2647	7.0081	8.0087	4.1996
Traditional machine learning	(9) Random forest	(10) Support vector machine	(11) Linear regression	(12) Logistic regression
	50.6241	93.7156	1083.1913	3828.8760
	(13) K-nearest neighbours	–	–	–
	122.6212	–	–	–
(14) Proposed method	1.0311	–	–	–

(1) Transformer; (2) RNN; (3) LSTM; (4) GRU; (5) 1D-CNN; (6) CNN-LSTM; (7) SAE-LSTM; (8) BiLSTM; (9)RF; (10) SVM; (11) LR; (12) LOR; (13) KN; (14) proposed method.

From Fig. 16, it can be seen that when using deep learning methods to analyze the dataset, the predicted RUL value deviates slightly from the true value and oscillates up and down around. It can be known from the RMSE results in Table 3, the deep learning model is much better for the analysis of RUL than the results obtained using traditional machine learning methods. Basically, the RMSE value of all those who use the deep learning methods to predict the RUL is less than 10. However, the results obtained by traditional machine learning methods in the same data settings are not satisfactory. The Transformer model has more oscillations, but the overall trend is around the actual RUL value. LSTM, RNN, and GRU have similar prediction results which are larger than the actual RUL value with GRU's prediction for the test set fluctuating more sharply in the second half. Among these three models used most frequently in RUL which are RNN, LSTM, and GRU, LSTM is the best. The result of RUL prediction using the one-dimensional CNN method is worse than LSTM, but the effect is slightly better when CNN is combined with LSTM whose RMSE is 7.0081 and the RMSE of LSTM is 7.1399. The BiLSTM used to test data has the best result which is 4.1996. After comparation, the PSR-former method is the most effective approach for bearing RUL prediction among those methods under the same data settings.

## Conclusions

Due to the complexity of the mechanical working environment, the data collected can be easily affected. A PSR-former remaining useful life prediction method was proposed in response to the situation. The contributions of this paper are summarized below:

1. To better reflect the degradation trend of the bearings, features chosen by the monotonicity were used to form a new enhanced HI index.
2. A PSR-former model was proposed including a PSR layer and a Transformer layer. PSR layer was used as an embedding to deepen the understanding of the characteristics. The Transformer was used to discriminate the RUL. A new layer-hopping was adopted in the self-attention structure in the PSR-former model to speed up the propagation and make the structure more stable.
3. The method was verified using IMS bearing dataset and compared with other deep learning methods and traditional machine learning algorithms. The effective implementation of the methods provides a theoretical basis for the data analysis in PHM.

However, the applicability of the model in other datasets needs further verification since this paper only analyzed the IMS bearing dataset. Time domain features were extracted in this manuscript to initially enhance the degradation trend and form a new health index. Other features in the time domain or frequency domain may also be extracted to build the degradation model, so it needs to be further explored in the future.

## Data Availability

The data that support the findings of this study are openly available in NASA Intelligrnt Systems Division at http://ti.arc.nasa.gov/tech/dash/pcoe/prognostic-data-repository/.
